# A social–ecological perspective on harmonizing food security and biodiversity conservation

**DOI:** 10.1007/s10113-016-1045-9

**Published:** 2016-09-26

**Authors:** Hannah Wittman, Michael Jahi Chappell, David James Abson, Rachel Bezner Kerr, Jennifer Blesh, Jan Hanspach, Ivette Perfecto, Joern Fischer

**Affiliations:** 1 Centre for Sustainable Food Systems and Institute for Resources, Environment and Sustainability, The University of British Columbia, 179-2357 Main Mall, Vancouver, BC V6T 1Z4 Canada; 2 Centre for Agroecology, Water and Resilience, Coventry University, Coventry, UK; 3 Institute for Agriculture and Trade Policy, Minneapolis, MN 55409 USA; 4 Faculty of Sustainability, Leuphana University Lueneburg, Scharnhorststrasse 1, 21335 Lueneburg, Germany; 5 Department of Development Sociology, Cornell University, Ithaca, NY 14850 USA; 6 School of Natural Resources and Environment, University of Michigan, Ann Arbor, MI 48109 USA

**Keywords:** Brazil, Cerrado, Food sovereignty, Food security, Land sparing, Land sharing, Sustainable intensification, Yield gaps

## Abstract

The major challenges of improving food security and biodiversity conservation are intricately linked. To date, the intersection of food security and biodiversity conservation has been viewed primarily through an agricultural “production lens”—for example, via the land sparing/sharing framework, or the concept of sustainable intensification. However, a productionist perspective has been criticized for being too narrow, and failing to consider other relevant factors, including policy, equity, and diversity. We propose an approach that conceptualizes rural landscapes as social–ecological systems embedded within intersecting multi-scalar processes. Based on such a framing, empirical research can be more clearly set in the context of system properties that may influence food security, biodiversity conservation, or both. We illustrate our approach through a description of contrasting agricultural systems within Brazil’s Cerrado region. We emphasize the need for new empirical research involving systematic comparisons of social–ecological system properties in landscapes threatened by food insecurity and ecosystem degradation.

**Electronic supplementary material:**

The online version of this article (doi:10.1007/s10113-016-1045-9) contains supplementary material, which is available to authorized users.

## Introduction

Two of the most pressing challenges of the twenty-first century are to improve global food security and more effectively conserve biodiversity (Tscharntke et al. 2012). Food security refers to a “situation that exists when all people, at all times, have physical, social and economic access to sufficient, safe and nutritious food that meets their dietary needs and food preferences for an active and healthy life” (FAO 2014). Food security is typically assessed according to dimensions of availability, economic and physical access, utilization (diet and nutrition), and stability (vulnerability and shocks). Biodiversity describes the diversity of genes, species, ecosystems, and their interactions (Convention on Biological Diversity 1992). Thus, biodiversity includes both wild and planned biodiversity and is most often assessed in terms of taxonomic, functional, and genetic richness and composition as well as their stability at both local and landscape levels.

Food security and biodiversity conservation are intimately connected, most obviously through agricultural production—which is widely recognized as a driver of biodiversity decline, but also a key factor in ensuring that sufficient food is available at any given scale (e.g., Godfray et al. 2010). On this basis, it is not surprising that many scientists have approached the intersection of food security and biodiversity conservation from a primarily production-oriented perspective. For example, an analytical framework focused on the relationship between the population densities of wild species and agricultural yields (often characterized as land sparing/sharing) has been proposed to investigate trade-offs between increasing agricultural production and biodiversity conservation (Green et al. 2005). Similarly, the notions of sustainable intensification and ecological intensification are primarily focused on pursuing increased production efficiency, while minimizing harm to (or even benefiting) biodiversity (Bommarco et al. 2013; Garnett et al. 2013). Both of these perspectives are motivated by a desire to meet global demand for food, which is increasing as a result of human population growth and dietary shifts in increasingly wealthy countries (e.g., China).

Despite their justified concern about meeting a rising demand for food, production-oriented perspectives have received two main criticisms. First, from a food security perspective, it is insufficient to focus on aggregate levels of production. In many instances, a lack of food production is not the main reason why people are food insecure; barriers to access and distribution—including poverty—often matter more (e.g., Sen 1984). Indeed, a recent comprehensive analysis of reductions in child malnutrition in developing countries between 1970 and 2010 found that only 18 % of the overall reduction could be attributed to increased yield (per capita dietary energy) and that increased per capita dietary energy was only the fourth strongest factor (out of six) for future reductions (Smith and Haddad 2015). Moreover, given that increased production, either through intensifying production or expansion of agricultural land, is generally assumed to cause ecosystem degradation and negatively impact biodiversity (Matson et al. 1997; Tilman 1999; Power 2010), this creates a potentially false dichotomy where food security and biodiversity conservation are assumed as competing “system goals” that must always involve trade-offs against each other. Conversely, there is evidence that biodiversity can actively contribute to food security (e.g., Frison et al. 2011; Burlingame and Dernini 2012; Smith and Haddad 2015; Table S1). For example, agricultural policies to improve food security outcomes may, indirectly and at times, contribute to biodiversity by tying program support to more sustainable production practices (Chappell et al. 2016; Wittman and Blesh 2015). Similarly, support for indigenous and traditional food systems as the basis for food security can also have a protective function for the maintenance of regional agrobiodiversity (van der Merwe et al. 2016; Barthel et al. 2013).

Second, both biodiversity conservation and food security are influenced by many variables beyond agricultural production (Loos et al. 2014; Fischer et al. 2014). For example, equity, empowerment, and good governance are important for both conservation (Speelman et al. 2014) and food security (Sonnino et al. 2014). As such, conservation programs must consider livelihood (and food security) impacts and provide good governance and incentives or compensation to ensure biodiversity protection (e.g., Scherr and McNeely 2008; Oldekop et al. 2016).

Third, current approaches to understanding the intersecting processes leading to food security and biodiversity outcomes often neglect explicit consideration of spatial and temporal scales, as well as the interactions between them (Fischer et al. 2014; Gibson et al. 2000). The over-simplification of trade-off models between food security and biodiversity can miss key mediating mechanisms such as community governance and other regulatory and policy environments (Lang et al. 2009), distributive and procedural justice (Loos et al. 2014) and diverse objectives of a broad range of actors in the food system (Ericksen 2008).

A focus on the impacts of agricultural production on biodiversity is important to ensure that long-term food availability is more ecologically sustainable, but says little about other important variables also affecting food security and biodiversity conservation. For example, individuals and particular groups can have limited rights and resources that limit their food security and/or can have negative impacts on biodiversity (Schipanski et al. 2016; Chappell and Lavalle 2011). A lack of attention to issues of equity and social justice can mean that increases in productivity can have no or even negative impacts on food security (Stone 2002). In summary, an integrated social–ecological systems approach is needed because agricultural landscapes are complex adaptive systems nested across scales, which affect both human well-being—including food security—and ecosystems (Liu et al. 2007).

To date, there is a lack of more holistic analytical approaches to address the linked concerns of food security and biodiversity conservation. Hence, there is a need for integrated assessment frameworks that include production considerations among a broader set of variables, including biophysical, social, and institutional dynamics across spatial scales (Fischer et al. 2014; Loos et al. 2014). To address this gap, we outline a conceptual approach where rural landscapes are viewed as social–ecological systems embedded within a spatial hierarchy of system properties that influence the food security–biodiversity conservation nexus. We emphasize that the purpose of our paper is not to suggest a specific solution to myriad challenges situated at this nexus. Rather, we seek to highlight important but under-recognized issues that researchers and practitioners can fruitfully engage with in the future.

We first propose a conceptual framework and suggest an initial list of system properties that both affect and are affected by biodiversity and food security. Second, we use this list to broadly characterize key system properties shaping two contrasting agricultural landscapes in the Cerrado region of Brazil. We highlight both similarities and differences in system properties between the two landscapes and link these differences to distinct outcomes related to food security and biodiversity conservation. We conclude by suggesting research priorities to further advance an interdisciplinary, systems-oriented perspective on food security and biodiversity conservation.

## A social–ecological systems perspective

Agricultural landscapes are characterized by complex interactions between social and ecological variables. We consider the landscape scale—including multiple ecosystems within a watershed or geo-politically defined area such as a region or municipality, and ranging in size from tens to hundreds of square kilometers—as a particularly useful unit of analysis for understanding challenges related to biodiversity conservation and food security, because it is meaningful from both ecological and social-institutional perspectives (Wu 2013; Fischer and Lindenmayer 2007). Moreover, landscapes can help to analytically integrate phenomena across multiple scales, because they are shaped by ecological and social dynamics at both smaller scales (e.g., patches or households), and the larger scales in which they are embedded (e.g., regions) (Selman 2006).

Although the social–ecological makeup of landscapes is shaped by many variables, it is often possible to identify a relatively small number of variables that are particularly influential with respect to particular outcomes. For example, Ostrom (2009) developed a general framework for analyzing the sustainability of social–ecological systems, identifying a sub-set of variables related to resources, governance systems, and users that have distinct interactions across resource system types and sizes. Her work demonstrated that more sustainable management of common property resources tended to be facilitated by a small number of system properties, such as effective ecological monitoring and governance arrangements that support collective decision-making, including attention to equity and accountability (Ostrom 2009, 421). In particular, these critical system properties are mediated by institutions. Defined as the rules, norms, and values governing a group of people, institutions can be formal (e.g., laws and official rules) or informal (e.g., cultural expectations and unwritten traditions). Both types of institutions can influence whether or not a given resource system is managed sustainably by a group of people, or collapses due to overexploitation and lack of cooperation.

The long-term goal of the framework proposed here is to identify a set of foundational system properties that benefit or hinder either food security or biodiversity conservation. In addition, we seek to identify leverage points for improving food security and biodiversity conservation outcomes through subsequent evaluations of the interactions between, and relative importance of, these properties. Ultimately, this is both a theoretical and empirical question requiring a major research effort. We hope to stimulate discussion on what a suitable “draft template” of important social–ecological system properties might look like, which can be refuted, adapted, or refined through future empirical work. Although it is impossible for any single empirical research project to give sufficient consideration to all relevant system properties, our template may offer an initial frame within which the broader implications, assumptions, and limitations of specific analyses can be contextualized.

**Fig. 1:**
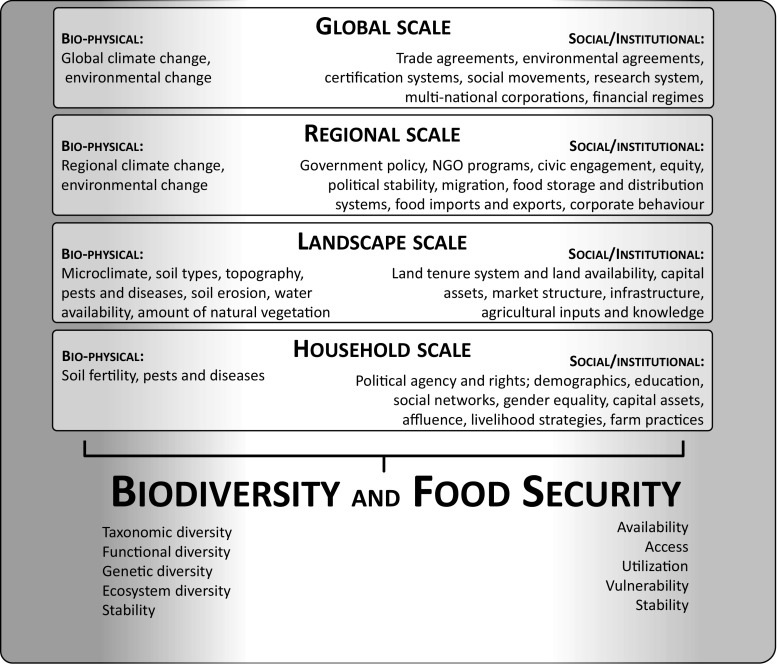
Schematic overview of social–ecological system properties at multiple scales that affect outcomes related to food security and biodiversity conservation (also see Table S1)

For convenience, we distinguish between biophysical and social-institutional system properties (Fig. 1; Table S1). At the landscape scale, among the potentially important biophysical properties shaping food security and biodiversity conservation outcomes are climate, soil types, topography, water availability, and the amount of native vegetation. Potentially important social properties include various forms of social and financial capital, social stratification, social networks and movements, political institutions, and gender relations, as well as governance-related aspects such as links to markets and infrastructure, land-tenure and resource distribution systems, and off-farm employment opportunities. Many of these factors are highly context dependent, meaning that the implications of a given land-management strategy will vary from place to place. For example, as we show below, even within the same region, contrasting socioeconomic and policy drivers that push distinct agricultural models may result in large differences in biodiversity and food security outcomes. As such, the “rules of the game” and interactional structures represented by institutions in particular regions strongly affect which aspects of biodiversity and food security are prioritized, by whom, and how, and thus can make the difference between ecosystem degradation and biodiversity conservation or between food security and widespread hunger.

In our conceptual model, system properties can be defined at multiple scales and also interact across scales. For example, from a biodiversity perspective, landscapes can be conceptualized as aggregations of patches, and the size and composition of these patches, and their connectivity, strongly shape landscape-level biodiversity outcomes. Similarly, from a food security perspective, landscapes contain numerous households that generate livelihood strategies based on accessing capital stocks (Fig. 1). System dynamics at larger or smaller scales also shape or constrain landscape-level outcomes. Larger-scale influences include shifting patterns of market demand and policy settings (including regulations and incentives), but also other formal and informal institutions, including community traditions, agrarian reform movements, NGO-led conservation programs, or certification schemes. Large-scale biophysical processes such as climate change can also influence and be influenced by landscape-level outcomes. There may also be reinforcing or dampening feedbacks between system properties across scales. For example, national agricultural policies can exacerbate (by creating institutional incentives for expansion of the agricultural frontier) or reduce biodiversity loss (by fostering and recognizing institutions supporting conservation) and also influence food security. Finally, interactions between institutions at different scales are key drivers of social–ecological outcomes (Ostrom 2009). For example, to enable the successful governance of a sustainable resource system, the locus of institutional power should be in local communities (at least in the established case of common property systems), with multiple, nested layers of coordinating institutions necessary for governing larger-scale systems (ESA 2013).

We emphasize that our conceptualization of rural landscapes as social–ecological systems (Fig. 1) is not intended to provide a blueprint for system classification. Rather, it is a starting point for more holistic, interdisciplinary research on linkages between food security and biodiversity conservation, which would extend and complement existing theoretical and empirical work on social–ecological systems and governance institutions. Similarly, it is worth noting that there is no single “correct” measure of either food security or biodiversity conservation—rather, how these are assessed (and at which scales) will depend on the specific case study at hand.

## Contrasting landscapes within Mato Grosso, Brazil

**Fig. 2:**
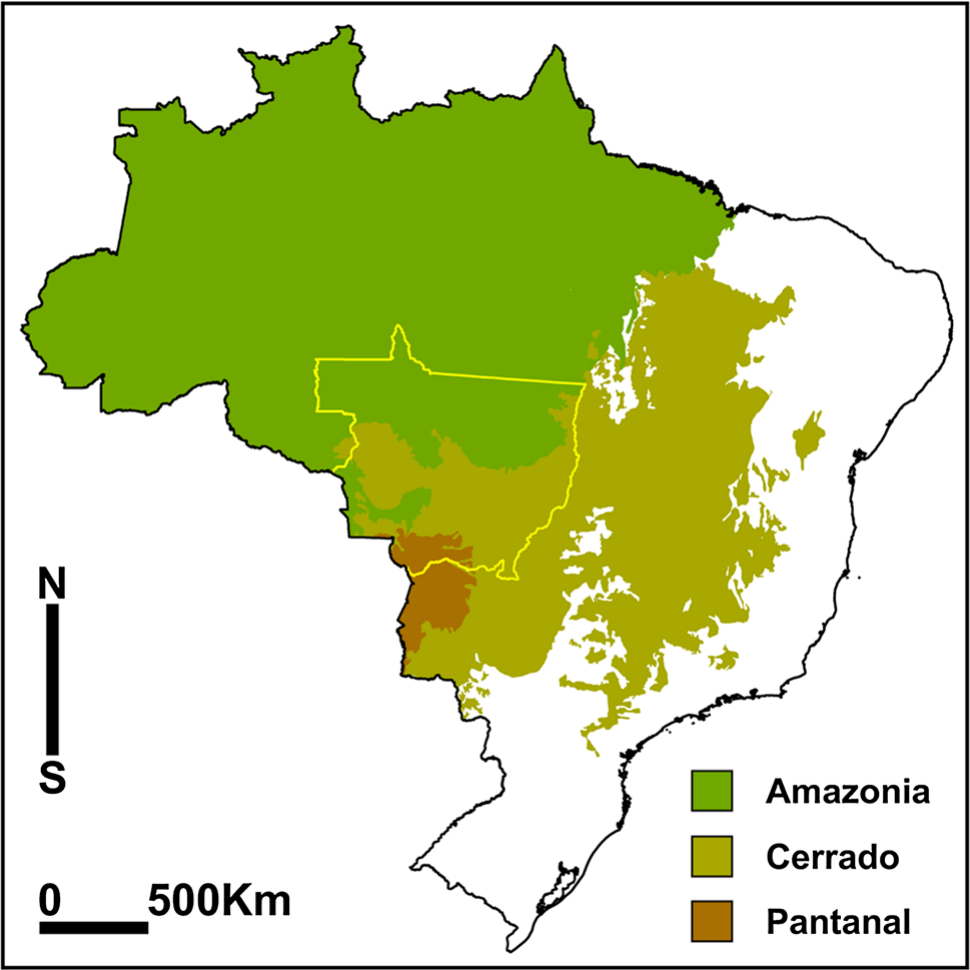
Mato Grosso (*outlined in yellow*) and its biomes (color figure online)

We illustrate our conceptual approach by contrasting two types of landscapes in the Brazilian Cerrado. Mato Grosso is Brazil’s third largest state (~900,000 km^2^), situated at the interface of tropical forest (Amazon), savannah/grassland (Cerrado) and wetland (Pantanal) biomes (Fig. 2). Biophysically, the Cerrado is characterized by highly weathered, acidic soils and a subtropical climate with distinct wet and dry seasons. Recent evidence suggests a lengthening dry season as a consequence of regional deforestation and climate change (Davidson et al. 2013). Mato Grosso is sparsely populated with 3.2 million residents mainly concentrated in the capital and the southern half of the state, and more than 50 % of GDP is generated by agricultural production. Despite rapid economic growth, 15 % of the population remains below the poverty line, and almost 20 % experienced food insecurity in 2013 (IBGE 2014). Mato Grosso’s agricultural transition has been a major contributor to widespread deforestation. Patterns of land occupation and clearing by colonist farmers vary from large-scale farms, including highly industrialized cropping systems and cattle ranches (some 42 million hectares), to smallholder family farms, which make up more than 80 % of the total number of farms, but are concentrated on 6.3 million hectares (IBGE 2009). Colonization of Mato Grosso’s agricultural frontier—as is the case in much of the Brazilian Amazon and Cerrado regions—was enabled by government land distribution and agrarian reform programs, with more capitalized farmers obtaining larger tracts of land conducive to mechanized production methods, and less capitalized farmers (i.e., the landless) receiving smaller plots of land in more remote regions (Simmons et al. 2010; Pacheco 2009).

Mato Grosso thus exhibits, among others, two types of contrasting landscapes that can be coarsely categorized as: (1) large-scale commodity production systems primarily characterized by beef and soybean exports, and requiring many external inputs including high-yielding seed varieties, chemical fertilizers, and pesticides; and (2) diversified smallholder family farms which focus on domestic consumer markets and commonly rely more heavily on locally available inputs including legumes and animal manures. The two landscape types share many common global and regional system properties (e.g., the same climatic conditions, regional and national governments, and regulatory frameworks). However, biophysical differences across farms and landscapes, differing formal and informal institutional characteristics, and the ways in which material, institutional, policy and regulatory resources are differentially accessed and used have resulted in distinct socioeconomic and ecological outcomes at the landscape and household levels across the two farming systems.

### Soybean landscapes

**Fig. 3:**
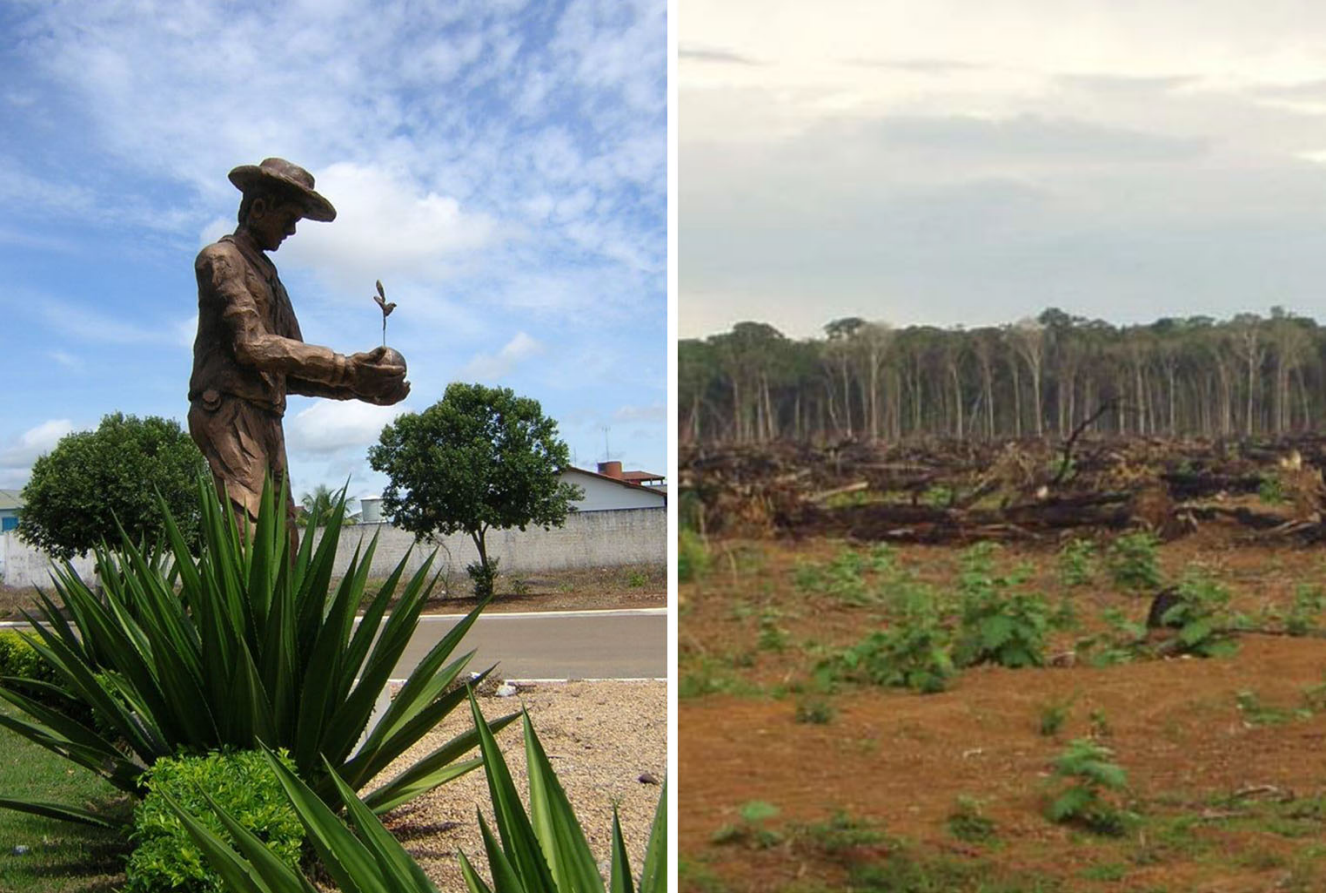
Investment in soybean landscapes has fostered economic growth for the global agribusiness sector and a small local population at the agricultural frontier, with local and possibly telecoupled costs to the environment

Mato Grosso’s contribution to Brazilian soybean production increased from 15 to 27 % between 1990 and 2010, with an average soybean farm measuring approximately 3000 ha. The expanding soybean landscape has been driven by regional, national and global system properties and institutional dynamics. By the 1970s, the Brazilian Agency for Agricultural Research (EMBRAPA), within the Brazilian Ministry of Agriculture, developed soybean varieties adapted to the Cerrado’s climate and soil types, and which are high yielding when grown with chemical inputs. In the 1980s, the federal government supported the migration of land-poor but moderately capitalized farmers from the southern regions of Brazil to the Cerrado through federal land distribution and colonization programs. Land titles were readily granted to soybean cooperatives, and public financing was made available to build supporting infrastructure, including export processing facilities and paved roads (VanWey et al. 2013). By the 1990s, private agribusiness research initiatives began developing their own locally adapted varieties, and multi-national investment in concentrated processing and export facilities further facilitated the expansion of the soybean frontier (Fig. 3).

At landscape and community scales, wealth from soybean production is typically concentrated among small numbers of producers and in agribusiness corporations, with relatively little investment in local economic development, the generation of more equitable livelihoods, or environmental sustainability (Garrett et al. 2013). The ability of a small number of people to maintain institutions favorable to their interests, and disrupt institutions that may in fact generate better collective outcomes for biodiversity and food security, reflects the common problem of “elite capture” and other inequalities in power and governance (e.g., Saunders 2014).

#### Biodiversity outcomes

The rapid expansion of industrialized agriculture in the Cerrado has been a major driver of biodiversity loss (Klink and Machado 2005). Between 2000 and 2005, Mato Grosso was responsible for the highest rates of deforestation in Brazil (Macedo et al. 2012). From 2006 to 2010, deforestation declined as soybean intensification occurred, but by 2010, 65 % of total 2010 deforestation in Mato Grosso was still directly attributable to soybean production (Lathuillière et al. 2014). In general, soybean farms are characterized by high external inputs, and relatively low biodiversity—noting, of course, that specific management practices and ecological impacts can vary widely across different environmental conditions and tenure regimes. These soybean cropping systems result in ecological feedbacks (e.g., declines in biodiversity and soil fertility) that further entrench dependence on fossil fuel-based inputs to sustain production. In addition, pesticide contamination of ground and surface waters in the Cerrado region is well documented (e.g., Laabs et al. 2002) with potential impacts for biodiversity and human and ecosystem health. Although Brazil’s national Forest Code requires legal forest reserves on 35 % of agricultural land in the Cerrado, the code has been weakly enforced, and voluntary zero-deforestation commitments such the Soy Moratorium are not in place for the Cerrado region (Gibbs et al. 2015; Soares-Filho et al. 2014). After the Forest Code was revised in 2012, many large-scale soybean farmers in Mato Grosso sought to compensate their deficit of legal reserves (due to historical deforestation) by participating in forest swapping schemes with properties in the neighboring, more forested, state of Pará, indicating that the impact of global commodity agreements such as the Soy Moratorium may simply shift deforestation from one region to another as export production continues to rise.

#### Food security outcomes

At the national and global levels, market conditions and increased global meat consumption—rather than local food security concerns—have been key drivers of soybean intensification and expansion. About half of the soybeans produced in Mato Grosso are exported internationally, with 66 % of total Brazilian soybean exports going to China in 2010, mainly destined for animal feed, and 20 % to the EU (Lathuillière et al. 2014). Allocating grains to animal feed is ecologically inefficient and reduces potential global food availability (Foley et al. 2011). National and international environmental agreements promoting biofuels have also driven expanding demand for soybean production, both for domestic use and export to Europe (Wilkinson and Herrera 2010). As such, expanded soybean production has led to increased availability and access to calories for distant global markets by supporting lower-cost meat production in China, and to soybean oil and animal feed for national consumption.

### Small-scale family farm landscapes

**Fig. 4:**
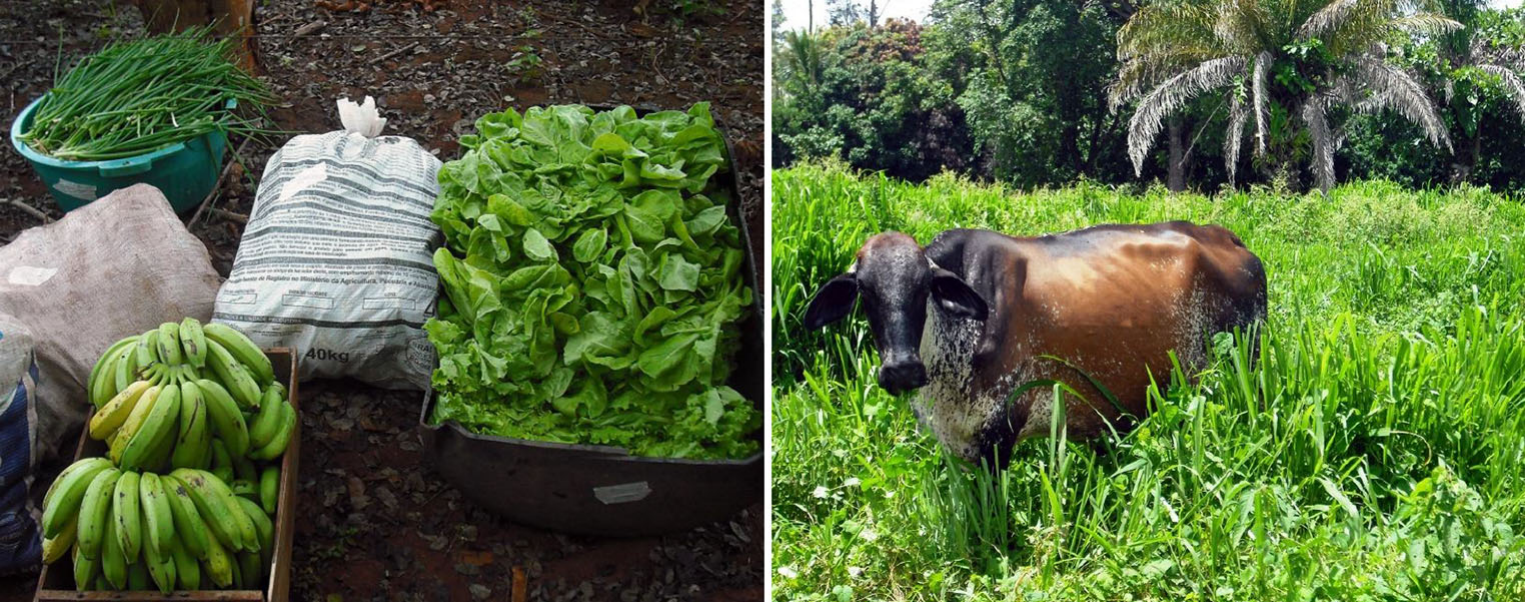
Small-scale family farms in the Cerrado utilize a diversified production model focusing on local markets and on-farm consumption, with a mix of field crops, small-scale dairy and beef production, and forest reserves

The small-scale family farm sector in Mato Grosso includes 85,000 farms averaging 30–70 ha, using primarily family labor to manage low external-input agricultural production systems oriented toward the domestic food economy (Fig. 4). These systems are characterized by mixed grain, vegetable, fruit, and livestock production, in addition to forest reserves. The family farm sector contributes 12 % to the Mato Grosso GDP and provides 4–5 jobs/100 hectares (compared to 0.3 jobs/100 hectares in mechanized agriculture) (de França et al. 2009).

The development of smallholder farms in Mato Grosso has been strongly influenced by social movements for agrarian reform within Brazil (Wittman 2009). These social movements are connected nationally and globally with groups that seek to organize more equitable access to rights and resources in support of sustainable rural livelihoods. This social mobilization has led to a strong landscape-level preference for farmer-led marketing cooperatives and the development of diversified local food economies. The focus on agricultural production for domestic consumption is supported by Brazil’s “Zero Hunger” initiative, which aims to increase food security by supporting rural livelihoods and more ecologically sustainable food production. “Zero Hunger” provides general support for the family farming sector, including targeted support for certified organic and agroecological diversification models, redistribution of agricultural credit to women and youth, and the re-development of local markets (Rocha 2009).

#### Biodiversity outcomes

Small-scale family farming communities are organized around an agricultural matrix in which diversified and low-input systems are integrated into the surrounding landscape. At a landscape level, both wild biodiversity (e.g., in forest reserves) and agrobiodiversity (e.g., mixed production of subsistence and market crops and livestock) are higher in small-scale family farm communities in the Cerrado when compared to the soybean production model (Godar et al. 2014). Notably, the reliable quantification of the biodiversity impacts of both soybean and family farming beyond Mato Grosso is currently not possible. This is both because of a lack of local studies, and because of an insufficient understanding of how the dynamics of land use, biodiversity degradation, and food security pathways in Brazil may affect other, distant locations via so-called teleconnections (see, for example, Liu et al. 2015).

#### Food security outcomes

Diversified production focusing on staple crops and vegetable crops, both for family subsistence and for sale in regional markets, is fundamental to improving Brazil’s domestic food security and household dietary diversity and quality (FAO 2014; Graeub et al. 2015). Domestic markets are more stable for family farmers than global export markets, because they are minimally affected by global price shocks and less susceptible to speculation (He and Deem 2010). At a household scale, engagement in diversified production of food crops for public procurement programs supports a more stable (though often low) household income and can improve household food availability, access, and utilization. A diversified mix of crops may also decrease vulnerability to economic (e.g., price volatility) and ecological (e.g., weather and pests) shocks by distributing risk across several crop types.

**Fig. 5:**
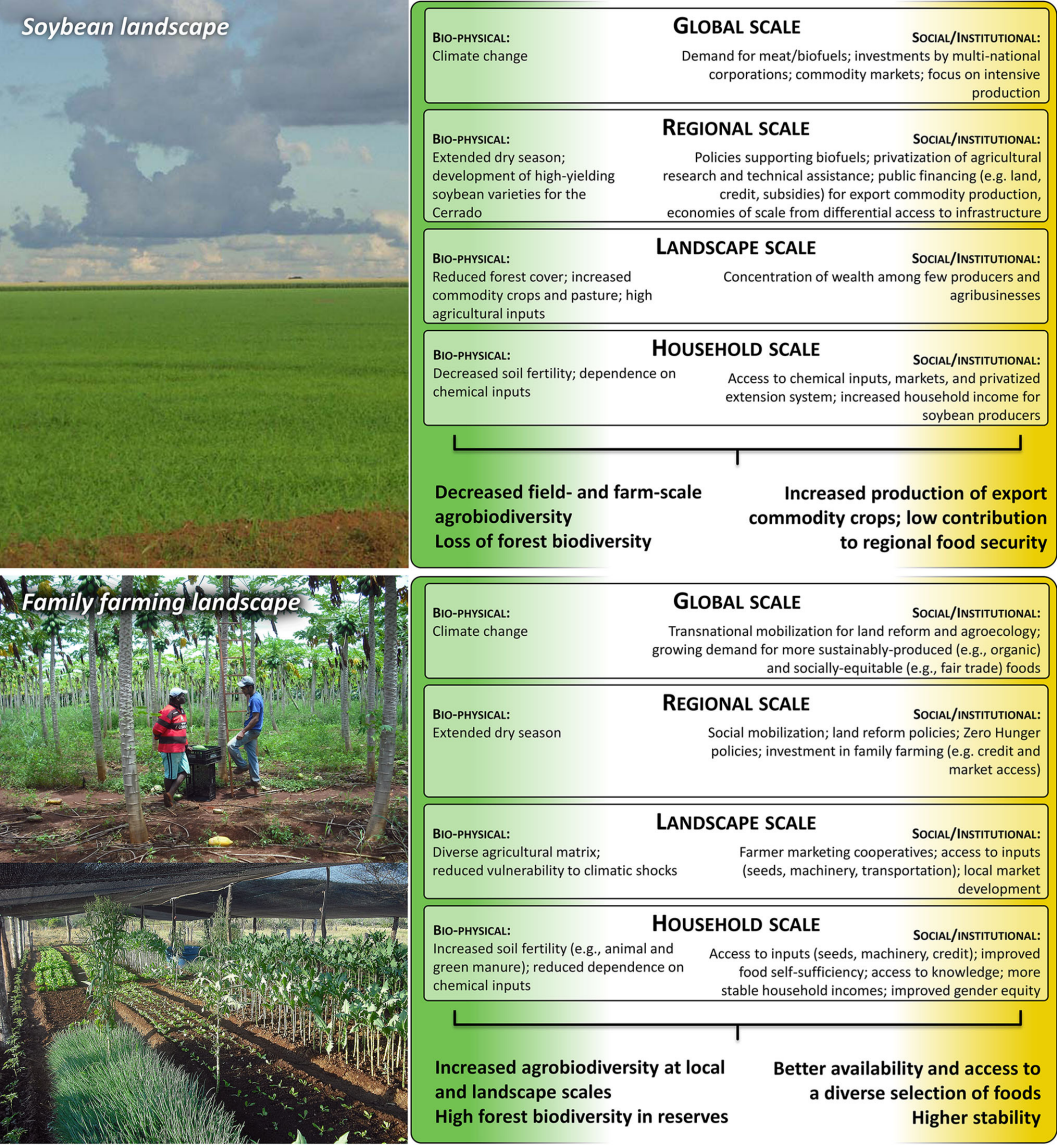
Stylized comparison between system biophysical and social-institutional properties shaping soybean landscapes and small-scale family farming landscapes in the Brazilian Cerrado. Soybean landscapes are characterized by low levels of biodiversity and low contributions to regional food security, and result from interactions between global resource demands, supportive biofuels policy, and poor environmental governance. Small-scale farming systems resulting from agrarian reform may also lead to deforestation, but tend to exhibit higher levels of agrobiodiversity and contribute to domestic food security

We highlight the multi-scalar interactions of elements of our conceptual framework across the two landscape types (Fig. 5) and highlight the results of differential access to and interactions with resources and institutions between farming systems.

### Implications of the case study

Our general characterization of two distinct agricultural systems in the Brazilian Cerrado serves to highlight three points. First, even when embedded within the same region, it is possible for landscapes to have very different system dynamics and interactions between social and ecological sub-systems. Second, such different properties are related to different outcomes for both food security and biodiversity conservation. This relationship is complex, and involves the interplay between many social and ecological variables across a range of scales. Third, a solely production-oriented analysis does not capture the complexity that defines the systems we compared.

The soybean landscape case study identifies the influence of systems properties at multiple scales, from global commodity booms to a poorly enforced Forest Code to differential access to land, technology, regional markets, processing and export infrastructure. These properties have created leverage points that constrain biodiversity conservation in the landscape, while making only a minimal contribution to regional food security. The family farming landscape, with greater agrobiodiversity and a regional agricultural mosaic contributing to domestic food markets, also responds to social–ecological drivers at multiple scales. These include the national food security policy *Fome Zero* which provides incentives for a transition to agroecological production (Wittman and Blesh 2015), and agrarian and environmental social movements that lobby for enforcement of the Forest Code and support for the family farm sector (Blesh and Wittman 2015).

## Further development of the proposed approach

The multi-scaled conceptual model that we provide here is a starting point for more systematic analyses of the complex and interactive biophysical and socio-institutional drivers of food security and biodiversity outcomes in particular landscapes—on its own, this approach does not allow conclusions as to which may be universal key factors or leverage points that facilitate improved outcomes in every case. The above analysis illustrates, however, that it is worthwhile to consider a wide range of social–ecological system properties and resulting outcomes for people and ecosystems—rather than singling out production-related variables such as yield as the primary metric of agricultural “performance.” To advance the conceptual approach outlined in this paper, we recommend three research priorities.

First, it would be useful to comprehensively and systematically review existing literature to ascertain which system properties have been shown to influence food security or biodiversity conservation, or both, and to understand the strength and nature of these influences. To this end, the list provided in Fig. 1 (and Table S1) is a starting point.

Second, we see an urgent need for major empirical research investigating the nexus of food security and biodiversity conservation through a more systems-based approach (e.g., Dougill et al. 2010; Ericksen 2008; Ericksen et al. 2009). As a starting point, it would be useful to conduct participatory workshops in a wide range of different landscapes to elicit relevant social–ecological system dynamics and analyze the social–ecological properties of these systems in relation to food security and biodiversity outcomes. This would provide a resource for comparing the underpinning system properties that determine food security and biodiversity conservation outcome across different systems. Existing work on a small number of cases suggests that certain constellations of system properties are likely to generate at least partly predictable outcomes with regard to food security and biodiversity conservation (e.g., Jackson et al. 2012).

Finally, it would be useful to accompany such broad-scale research with in-depth social–ecological studies in selected rural landscapes that are potentially food insecure and contain at-threat biodiversity. Much existing research on the intersection of food and biodiversity has been analytically sophisticated, but has not accounted for the multifaceted and complex nature of real-world social–ecological systems. In-depth case studies and fieldwork provide a valuable “reality check” for the insights generated via broader cross-system comparisons and may identify leverage points for scaling up systems that generate the greatest co-benefits for biodiversity and food security.

Crucially, the approach outlined here does not represent an end point of this research nexus. A further necessary step will be to understand how the system properties within agricultural landscapes, as well as urban centers, interact and shape biodiversity and food security outcomes across multiple spatial and temporal scales. We hope that the conceptual approach suggested in this paper will stimulate much-needed discussion as well as new empirical research on how to best address two of the most urgent problems of our times.

## Electronic supplementary material

Below is the link to the electronic supplementary material.

Supplementary material 1 (PDF 799 kb)
