# Supplementary material for: A social–ecological perspective on harmonizing food security and biodiversity conservation
Source: Reg Environ Change. 2016 Sep 26;17(5):1291–301. doi: 10.1007/s10113-016-1045-9 (PMC6979715; doi:10.1007/s10113-016-1045-9)
Supplement: Supplementary file 1 — Supplementary material 1 (PDF 799 kb) [file 10113_2016_1045_MOESM1_ESM.pdf]

Properties	***Outcome affected***	***Mechanism/Relationship***	***Example Sources***
**Bio-physical properties**			
Global climate change; environmental change	Food security	Challenges to farmer livelihoods; effects on yield; uncertainty and instability in production, food supply, and food prices.	Calzadilla et al. 2013; Porter et al. 2014; World Bank 2007; Ringler et al. 2010
	Biodiversity	Multiple direct and indirect effects on species habitats, ranges, stressors, and extinction risks.	Staudt 2013; Fordham et al. 2011; Klausmeyer et al. 2011; Huston 2005
Soil types and fertility, soil erosion, topography	Food security	Direct effect on productivity, indirect effects on production costs and market prices; soil degradation, particularly reduced soil organic matter, affects quantity and quality of food production (e.g., increases susceptibility to drought stress and nutrient deficiencies, and increases susceptibility to pest and disease outbreaks). Soil contamination directly affects food quality (e.g. arsenic in rice) and human health.	Lal 2009; Khan et al. 2010; Scherr 1999
	Biodiversity	Biodiversity influenced directly via soil quality feedbacks on belowground biodiversity and the soil microbiome, and indirectly through soil fertility effects on net primary productivity, and/or increased fertilizer use to maintain yields on degraded soils.	Postma-Blaauw 2010; McDaniel et al. 2014; Tilman et al. 1996; Mozumder and Berrens 2007
Water availability (and safety)	Food security	Strong contributor to malnutrition reduction (as indicator of overall health environment); importance in agricultural production.	Hanjra and Qureshi 2010; Smith and Haddad 2015; Armah et al. 2011; Turral et al. 2011; Khan et al. 2010
	Biodiversity	Agricultural impacts on hydrologic cycles and water quality can directly threaten biodiversity.	Zedler 2003; Geng et al. 2015; Gleick 1998
Amount and diversity of natural vegetation	Food security	Connected to dietary diversity and wild collection; provides ecosystem services to agriculture (e.g., pollination, pest control).	Belanger and Johns 2008; Chappell et al. 2013; Lira et al. 2009; Power 2010
	Biodiversity	Forest degradation and fragmentation leads to loss of wild biodiversity.	Godar et al. 2015; Savilaasko et al. 2013; Melo et al. 2013;

			Grau et al. 2013; Fearnside 2005
Agrobiodiversity	Food security	Dietary diversity tied very strongly to food security directly, and to nutritional quality of diets such as reductions in hidden hunger/micronutrient deficiencies, as well as to decreased risk of crop failure and increased ecosystem services (see also references for diversity in natural vegetation).<br><br>Crop diversity can increase the stability and reduce vulnerability of both agricultural yields and farm incomes in the face of both market and biophysical perturbations to farming systems. The stabilizing/risk reducing outcome of increased agrobiodiversity (from within-crop genetic diversity, to diversified cropping patterns), is likely to be of increasing importance with increasing climate and market instability.	Abson et al. 2013; Belanger and Johns 2008; Burlingame and Dernini 2012; Di Falco and Perrings 2003; Di Falco and Chavas 2006; Di Falco and Chavas 2009; Di Falco et al. 2010; Ericksen 2008; Fraser 2003; Frison et al. 2011; Johns and Eyzaguirre 2006; Liebman and Schulte 2015; Smith and Haddad 2015; Zimmerer 1998
	Biodiversity	A positive association between planned (agrobiodiversity) and associated ("wild") biodiversity has been, according to Vandermeer et al. (2002), established "beyond credible doubt" for vertebrates, arthropods, and non-crop plants.	Liebman and Schulte 2015; Vandermeer et al. 2002
Pests and diseases	Food Security	Increased pest and disease pressure directly reduces crop yields.	Verberg et al. 2013; Matson et al. 1997
	Biodiversity	Wild biodiversity and agrobiodiversity reduce pest and disease pressure (e.g., by providing habitat for natural enemies, or by serving as "trap crops" for pests). Soil microbial diversity can suppress diseases.	Barthel et al. 2013; Bommarco et al. 2013; Matson et al. 1997; Garbeva et al. 2004
**Social properties**			
Trade agreements	Food security	Highly contingent; often thought to be mediated via economic growth and access to cheaper food; however, connections between food prices and food security are contested<br><br>Inequity and lack of appropriate redistribution within national contexts can hinder or eliminate theorized food security gains from international agricultural trade practices.	Brown et al. 2014; FAO 2012; Wise 2009; Weis 2007; Tansey and Rajotte 2008;<br>Haddad 2015; Otero et al. 2013; Heady 2010

	Biodiversity	Complex; land-displacement literature growing; international trade is increasing invasive species.	Lenzen et al. 2012; Meyfroidt et al. 2013; Bax et al. 2003
Environmental agreements	Food security	The focus of REDD+, CBD, and Kyoto on increasing forest cover may reduce agricultural area and productivity; inclusion of agricultural soil carbon sequestration contracts can raise income and improve food security.	FAO 2013a; Antle et al. 2009; Corson and Macdonald 2012
	Biodiversity	Increase in conservation area may improve preservation of wild biodiversity; appropriate scale and community engagement needed for effective governance.	Hodge and Adams 2014; Ewers et al. 2009; Brannstrom 2001; McAfee and Shapiro 2010
Certification systems	Food security & biodiversity	Fair trade: documented multiple effects on farming systems, biodiversity, livelihoods, and food security; effects vary with the social and political institutions regulating fair trade schemes.	Bacon et al. 2008; Jaffee 2007; Jaffee and Howard 2009; Raynolds 2000
Financial regimes and multinational corporations	Food security	Investment and speculation can affect food prices and livelihoods.	Davis 2001; De Schutter 2010; IATP 2008
	Biodiversity	Largely speculative as a “financialization of biodiversity” is still in developmental and uncertain stage; could be mediated through investments in offsets and finances of conservation.	Doswald et al. 2012; Phelps et al. 2011; McAfee 1999
Research system	Food security	Privatization of agricultural research reduced support for research on low-input agricultural practices and subsistence models	IAASTD 2009; Sumberg et al. 2012a, b; Levidow et al. 2014
	Biodiversity	Determinants of innovation within agricultural research systems have led to technological systems favoring specialized, low-diversity agroecosystems.	Vanloqueren and Baret, 2009
Government policy	Food security	Many possible avenues of effect through effects on entitlements and underlying determinant variables.	da Silva et al. 2011; Lappé et al. 2013; Smith and Haddad 2000; 2015; Rocha 2009; Wise 2004
	Biodiversity	Affected directly by conservation policies and indirectly by many other policies (including agricultural policies).	Ceddia et al. 2013; Chopra et al. 2005; Soares-Filho et al. 2014
NGO programs social	Food security	Multi-faceted and variable ways in which civic engagement and civil society organizations can influence food security both	Abebaw et al. 2010; Seed et al. 2013; Wittman and

movements and civic engagement		positively and negatively. Can play a crucial role in mobilizing underprivileged groups to advocate for greater rights and increased access.	Blesh, 2015.
	Biodiversity	Social movements can play a crucial role in promoting biodiversity in regions of high inequality.	Perfecto and Vandermeer 2008; Wittman 2010
Equity and justice	Food security	Affects distribution, political effectiveness, access rights, and multiple other factors.	Haddad 2015; Friel and Baker 2009; Sen 1981; Sievers-Glotzbach 2014
	Biodiversity	Driving mechanisms/ underlying correlates unclear.	Holland et al. 2009; Mikkelson et al. 2007
Political stability	Food security	Instability and conflict affects many elements of food security, from food supply to entitlements and rights.	FAO 2000; Ó Gráda 2009
	Biodiversity	Possible links little-explored; legacy of conflicts may have profound indirect effects.	Russell 2001; Smith et al. 2003; Hamilton et al. 2000
Migration and Demographics	Food security	Rural out-migration increases dependency on imported food subject to global price shocks; urbanization and changing food preferences affect global demand and supply.	Otero 2011; de Janvry and Sadoulet 2010; Regmi and Meade 2013
	Biodiversity	Habits of urban dwellers will highly influence biodiversity outcomes.	CBD 2012; McSweeney 2005
Food storage and distribution systems (imports/exports)	Food security	Grain reserves aim to address food price volatility associated with food imports and exports.	Murphy 2009; Gilbert 2011; Wright 2009; Brigham 2011; Headey 2010
	Biodiversity	Increased reliance on food imports may reduce pressure to expand agricultural land base, but increase deforestation in other regions.	Walker 2014; Melo et al. 2013; DeFries et al. 2010
Land tenure system and land availability	Food security	Food security depends on adequate land access for smallholder and domestic food supply systems.	HLPE 2013; Borras 2003; 2010; 2012; White et al. 2012; FAO 2013b; Young 1999; Assies 2009
	Biodiversity	Property rights regimes provide both structure and incentives for natural resource use and conservation.	Hodge and Adams 2014; Ostrom et al. 1999; McKean 2000; Merenlander et al. 2004; Brannstrom 2001; Wittman

			2009, 2010.
Access to infrastructure and agricultural inputs	Food security	Market and distribution infrastructure and access to agricultural inputs shape production systems, food system resilience, and food accessibility.	World Bank 2007; Sumberg et al. 2012b; IAASTD 2009; Patel et al. 2014; Bezner Kerr 2012; 2005
	Biodiversity	High input agricultural systems, especially at the agricultural frontier involving land clearing, impact biodiversity and landscape degradation; road infrastructure can shape advancement of the agricultural frontier.	Baletti 2012; Fearnside 2001; Matson et al. 1997; Barona et al. 2010
Political agency and rights	Food security	Citizen role in setting food policy affects food availability and distribution systems.	Edelman and Carwil 2011; Edelman 2008; Borras et al. 2008; Wittman et al. 2009; Wittman 2011
	Biodiversity	Political and social entitlements shape access and use of environmental resources and services.	Leach et al. 1999; Wittman et al 2010; Wittman and Blesh 2015
Education, Knowledge and Social Networks	Food security	Multiple benefits, including possible increases in agricultural productivity, agrobiodiversity, entitlements, maternal, and postpartum care, agency, nutritional knowledge.	Smith and Haddad 2015; Alderman and Headey 2014; Nuñez-Espinoza et al. 2014; Wittman and Blesh, 2015
	Biodiversity	Alters the normative underpinnings for biodiversity conservation; may increase agrobiodiversity and agroecological management practices.	Van Weelie and Wals 2002; García-Barrios et al. 2008; McAfee and Shapiro 2010; Wittman et al 2010
Gender equity/women's status	Food Security	Multiple benefits, including possible increases in agricultural productivity, entitlements, maternal, and postpartum care, agency, nutritional knowledge; increased say in household spending; increased productivity from equal access to resources	Alderman and Headey 2014; Agarwal 2015; Smith and Haddad 2000; 2015

	Biodiversity	Greater gender equality can play a critical role in maintaining biodiversity in forest ecosystems; alters the normative underpinnings for biodiversity conservation.	Van Weelie and Wals 2002; García-Barrios et al. 2008; Agarwal 2009; 1997; 1988; McSweeney 2005; Zimmerer et al. 2015
Affluence and capital assets	Food security	Income has multiple indirect effects on food security, as well as serving as a form of food entitlement.	Sen 1981; Smith and Haddad 2000; 2015
	Biodiversity	Affluence drives biodiversity-harming consumption through a variety of mechanisms.	Bradshaw et al. 2010; Holland et al. 2009; Weinzettel et al. 2013
Farm practices	Food security	Crop choice, diversification and farming type (for subsistence, local markets, and export) affect household and community food availability and price.	Seufert et al. 2012; Badgley et al. 2007; Connor, 2007; Dahal et al. 2009; Kasem and Thapa, 2011; Jones 2015; Jones et al 2014; Blesh and Wittman, 2015
	Biodiversity	Land management decisions, including deforestation at the agricultural frontier, affect both wild and on-farm biodiversity, particularly with negative effects of high input agricultural practices; crop rotation selection, use of organic nutrient amendments, reduced chemical inputs, and building soil organic matter reserves all impact planned and associated biodiversity.	Chappell et al. 2013; Frishkoff et al. 2014; IAASTD 2009; Power 2010; Norton et al. 2013; Phelps et al. 2013; Barona et al. 2010; Jarvis 2008; Blesh and Wittman, 2015

Literature cited

Abebaw D, Fentie Y, and Kassa B. 2010. The impact of a food security program on household food consumption in Northwestern Ethiopia: A matching estimator approach. *Food Policy* ***35***: 286–293.

Abson DJ, Fraser EDG, and Benton TG. 2013. Landscape diversity and the resilience of agricultural returns: a portfolio analysis of land-use patterns and economic returns from lowland agriculture. *Agriculture & Food Security*, **2**: 1-15.

Agarwal B. 2015. Food Security, Productivity, and Gender Inequality. In: Herring RJ (Ed). *The Oxford Handbook of Food, Politics, and Society*. Oxford: Oxford University Press.

Agarwal B. 2009. Gender and forest conservation: The impact of women's participation in community forest governance. *Ecological Economics* **68**: 2785–99.

Agarwal B. 1997. Gender, Environment, and Poverty Interlinks: Regional Variations and Temporal Shifts in Rural India, 1971-91. *World Development* **25**: 23–52.

Agarwal B. 1988. Neither Sustenance nor Sustainability: Agricultural Strategies, Ecological Degradation and Indian Women in Poverty. In: Agarwal B (Ed). *Structures of Patriarchy*. New Delhi: Kali for Women.

Alderman H and Headey DD. 2014. *The Nutritional Returns to Parental Education*. International Food Policy Research Institute, Washington, D.C.

Antle JM and Stoorvogel JJ. 2008. Agricultural carbon sequestration, poverty, and sustainability. *Environment and Development Economics* **13**: 327–52.

Armah FA, Odoi JO, Yengoh GT, *et al.* 2011. Food security and climate change in drought-sensitive savanna zones of Ghana. *Mitigation and Adaptation Strategies for Global Change* **16**: 291-306.

Assies W. 2009. Land tenure, land law and development: some thoughts on recent debates. *Journal of Peasant Studies* **36**: 573–89.

Bacon CM, Mendez VE, Gliessman, SR, *et al.* (Eds). 2008. *Confronting the coffee crisis: Fair Trade, sustainable livelihoods and ecosystems in Mexico and Central America*. Cambridge, MA: MIT Press.

Badgley C, Moghtader J. Quintero E, *et al.* 2007. Organic agriculture and the global food supply. *Renew Agr Food Syst* **22**: 86–108.

Baletti B. 2012. Ordenamento Territorial: Neo-developmentalism and the struggle for territory in the lower Brazilian Amazon. *Journal of Peasant Studies* **39**: 573.

Barona E, Ramankutty N, Hyman G, and Coomes OT. 2010. The role of pasture and soybean in deforestation of the Brazilian Amazon. *Environmental Research Letters* **5**: 024002.

Barthel S, Crumley C, and Svedin U. 2013. Bio-cultural refugia—Safeguarding diversity of practices for food security and biodiversity. *Global Environmental Change* ***23***: 1142–52.

Bax N, Williamson A, Aguero M, *et al.* 2003. Marine invasive alien species: a threat to global biodiversity. *Marine policy* **27**: 313-23.

Bélanger J and Johns T. 2008. Biological diversity, dietary diversity, and eye health in developing country populations: establishing the evidence-base. *EcoHealth* **5**: 244–56.

Bezner Kerr R. 2005. Food Security in Northern Malawi: Gender, Kinship Relations and Entitlements in Historical Context. *Journal of Southern African Studies* **31**: 53–74.

Bezner Kerr R. 2012. Lessons from the old Green Revolution for the new: Social, environmental and nutritional issues for agricultural change in Africa. *Progress in Development Studies* **12**: 213–29.

Blesh J and Wittman H. 2015. "Brasilience:" Assessing Resilience in Land Reform Settlements in the Brazilian Cerrado. *Human Ecology*. **43**: 531-546. 10.1007/s10745-015-9770-0

Bommarco R, Kleijn D, and Potts SG. 2013. Ecological intensification: harnessing ecosystem services for food security. *Trends in Ecology and Evolution* ***28***: 230–38.

Borras SM and Franco JC. 2010. Food Sovereignty and Redistributive Land Policies: Exploring Linkages, Identifying Challenges. In: Wittman H, Desmarais AA and Wiebe N (Eds). *Food Sovereignty: Reconnecting Food, Nature and Community*. Halifax: Fernwood Publishing.

Borras SM Jr. 2003. Questioning Market-Led Agrarian Reform: Experiences from Brazil, Colombia and South Africa. *Journal of Agrarian Change* **3**: 367–94.

Borras SM Jr, Edelman M, and Kay C. 2008. Transnational agrarian movements: origins and politics, campaigns and impact. *Journal of Agrarian Change* **8**: 169–204.

Borras SM Jr and Franco JC. 2012. Global Land Grabbing and Trajectories of Agrarian Change: A Preliminary Analysis. *Journal of Agrarian Change* **12**: 34–59.

Bradshaw CJA, Giam X, Sodhi NS. 2010. Evaluating the Relative Environmental Impact of Countries. *PLoS ONE* **5**: e10440.

Brannstrom C. 2001. Conservation-with-development models in Brazil's agro-pastoral landscapes. *World Development* **29**: 1345–59.

Brigham AM. 2011 Agricultural Exports and Food Insecurity in Sub-Saharan Africa: A Qualititative Configurational Analysis. *Development Policy Review* **29**: 729-48.

Brown C, Murray-Rust D, van Vliet J, *et al.* 2014. Experiments in Globalisation, Food Security and Land Use Decision Making. *PLoS ONE* **9**: e114213.

Burlingame B and Dernini S (Eds). 2012. *Sustainable Diets and Biodiversity: Directions and solutions for policy, research and action*. Rome, Food and Agriculture Organization of the United Nations.

Calzadilla A, Rehdanz K, Betts R, *et al*. 2013. Climate change impacts on global agriculture. *Climatic Change* **120**: 357-74.

Ceddia MG, Sedlacek S, Bardsley NO, and Gomez-y-Paloma S. 2013. Sustainable agricultural intensification or Jevons paradox? The role of public governance in tropical South America. *Global Environmental Change* **23:** 1052-1063.

Chappell MJ, Wittman H, Bacon CM, *et al.* 2013. Food sovereignty: an alternative paradigm for poverty reduction and biodiversity conservation in Latin America. *F1000Research* [v1; ref status: indexed, http://f1000r.es/23s] *F1000Research* **2**:235

Chopra K, Leemans R, Kumar P and Simons H (Eds). 2005. *Ecosystems and Human Well-being: Policy Responses, Volume 3: Findings of the Responses Working Group of the Millennium Ecosystem Assessment*. Washington, D.C.: Island Press.

Connor DJ. 2007. Organic agriculture cannot feed the world. *Field Crops Research* **106**: 187–90.

Corson C and MacDonald KI. 2012. Enclosing the global commons: the convention on biological diversity and green grabbing. *Journal of Peasant Studies* **39**: 263–83.

CBD (Convention on Biological Diversity). 2012. *Cities and biodiversity outlook: Action and policy: A global assessment of the links between urbanization, biodiversity, and ecosystem services*. Secretariat of the Convention on Biological Diversity.

da Silva JG, Del Grossi ME, and de França CG (Eds). 2011. *Fome Zero (Zero Hunger Program): The Brazilian experience*. Brasilia: MDA/FAO.

Dahal BM, Nyborg I, Sitaula BK and Bajracharya RM. 2009. Agricultural intensification: food insecurity to income security in a mid-hill watershed of Nepal. *International Journal of Agricultural Sustainability,* **7**: 249-60.

Davis M. 2001. *Late Victorian holocausts: El Niño famines and the making of the third world*. London: Verso.

de Janvry A and Sadoulet E. 2010. The Global Food Crisis and Guatemala: What Crisis and for Whom? *World Development* **38**: 1328–39.

De Schutter O. 2010. *Food Commodities Speculation and Food Price Crises: Regulation to reduce the risks of price volatility*. United Nations. Louvain-la-Neuve, Belgium.

DeFries RS, Rudel T, Uriarte M, and Hansen, M. 2010. Deforestation driven by urban population growth and agricultural trade in the twenty-first century. *Nature Geoscience* **3**: 178–81.

Di Falco S, Bezabih M and Yesuf M. 2010. Seeds for livelihood: Crop biodiversity and food production in Ethiopia. *Ecological Economics* **69**: 1695-1702.

Di Falco S and Chavas J-P. 2006. Crop genetic diversity, farm productivity and the management of environmental risk in rainfed agriculture. *European Review of Agricultural Economics* **33**: 289-314.

Di Falco S and Chavas J-P. 2009. On crop biodiversity, risk exposure, and food security in the highlands of Ethiopia. *American Journal of Agricultural Economics* **91**: 599-611.

Di Falco S and Perrings C. 2003. Crop genetic diversity, productivity, and stability of agroecosystems. A theoretical and empirical investigation. *Scottish Journal of Political Economy* **50**: 207-16.

Doswald N, Barcellos Harris M, Jones M, Pilla E, and Mulder I. 2012. *Biodiversity offsets: voluntary and compliance regimes. A review of existing schemes, initiatives and guidance for financial institutions*. Cambridge, UK and Geneva, Switzerland: UNEP-WCMC and UNEP FI.

Edelman M. 2008. Transnational Organizing in Agrarian Central America: Histories, Challenges, Prospects. *Journal of Agrarian Change* **8**: 229–57.

Edelman M and Carwil J. 2011. Peasants' rights and the UN system: quixotic struggle? Or emancipatory idea whose time has come? *Journal of Peasant Studies* **38**: 81–108.

Ericksen PJ. 2008. What is the vulnerability of a food system to global environmental change. *Ecology and Society* **13**: 14.

Ewers RM, Scharlemann JPW, Balmford A and Green RE. 2009. Do increases in agricultural yield spare land for nature? *Glob Change Biol* **15**: 1716–26.

FAO (Food and Agriculture Organization of the United Nations). 2013a. A guide to Agriculture at UNFCCC COP 19 (Warsaw, Poland, 11-22 November 2013). Rome: FAO.

FAO (Food and Agriculture Organization of the United Nations). 2013b. The State of Food Insecurity in the World: the multiple dimensions of food insecurity. Rome: FAO.

FAO (Food and Agriculture Organization of the United Nations). 2012. Regional trade agreements and food security in Asia. Bangkok: FAO.

FAO (Food and Agriculture Organization of the United Nations). 2000. The state of food and agriculture 2000. Rome: FAO.

Fearnside PM. 2001. Soybean cultivation as a threat to the environment in Brazil. *Environ Conserv* **28**: 23–38.

Fearnside PM. 2005. Deforestation in Brazilian Amazonia: History, rates, and consequences. *Conservation Biology* **19**: 680–8.

Fordham DA, Wigley TM, and Brook B. 2011. Multi-model climate projections for biodiversity risk assessments. *Ecological Applications* ***21***: 3317–31.

Fraser EDG. 2003. Social vulnerability and ecological fragility: building bridges between social and natural sciences using the Irish Potato Famine as a case study. *Conservation Ecology* **7**: 9.

Friel S and Baker PI. 2009. Equity, food security and health equity in the Asia Pacific region. *Asia Pacific journal of clinical nutrition* **18**: 620-32.

Frishkoff LO, Karp DS, M'Gonigle LK *et al.* 2014. Loss of avian phylogenetic diversity in neotropical agricultural systems. *Science* **345**: 1343-6.

Frison EA, Cherfas J, and Hodgkin T. 2011. Agricultural Biodiversity Is Essential for a Sustainable Improvement in Food and Nutrition Security. *Sustainability* **3**: 238–53.

Garbeva P, Van Veen J and Van Elsas J. 2004. Microbial diversity in soil: selection of microbial populations by plant and soil type and implications for disease suppressiveness. *Annual Review of Phytopathology* **42**: 243-70.

García-Barrios L, Speelman EN and Pimm M. 2008. An educational simulation tool for negotiating sustainable natural resource management strategies among stakeholders with conflicting interests. *Ecological Modeling* **210**: 215-26

Geng SM, Yan DH, Zhang TX, *et al.* 2015. Effects of drought stress on agriculture soil. *Natural*

Hazards **75**: 1997-2011.

Gilbert C. 2011. *Food reserves in developing countries: Trade policy options for improved food security* (Issue Paper No. 37). Geneva: International Centre for Trade and Sustainable Development.

Gleick PH. 1998. Water in crisis: paths to sustainable water use. *Ecological Applications* ***8:*** 571–9.

Godar J, Persson UM, Tizado EJ, and Meyfroidt P. 2015. Towards more accurate and policy relevant footprint analyses: Tracing fine-scale socio-environmental impacts of production to consumption. *Ecological Economics* **112**: 25–35.

Grau R, Kuemmerle T and Macchi L. 2013. Beyond "land sparing versus land sharing": environmental heterogeneity, globalization and the balance between agricultural production and nature conservation. *Current Opinion in Environmental Sustainability* ***5***: 1–7.

Haddad L. 2015. Equity: Not only for idealists. *Development Policy Review* **33**: 5-13.

Hamilton A, Cunningham A, Byarugaba D and Kayanja F. 2000 Conservation in a region of political instability: Bwindi Impenetrable forest, Uganda. *Conservation Biology* **14**: 1722-5.

Hanjra MA and Qureshi ME. 2010. Global water crisis and future food security in an era of climate change. *Food Policy* **35:** 365-77.

Headey D. 2010. *Rethinking the Global Food Crisis: The Role of Trade Shocks*. International Food Policy Research Institute, Washington, D.C.

HLPE (High Level Panel of Experts). 2013. I*nvesting in smallholder agriculture for food security*. Rome: The High Level Panel of Experts on Food Security and Nutrition of the Committee on World Food Security.

Hodge ID and Adams WM. 2014. Property institutions for rural land conservation: Towards a post- neoliberal agenda. *Journal of Rural Studies* **36**: 453–62.

Holland TG, Peterson GD and Gonzalez A. 2009. A Cross-National Analysis of How Economic Inequality Predicts Biodiversity Loss. *Conservation Biology* **23**: 1304–13.

Huston MA. 2005. The three phases of land-use change: implications for biodiversity. *Ecological Applications* **15**: 1864–78.

IAASTD (International Assessment of Agricultural Knowledge Science and Technology for Development). 2009. *Agriculture at a crossroads: International assessment of agricultural knowledge, science and technology for development*. Washington, D.C.: Island Press.

IATP (Institute for Agriculture and Trade Policy). 2008. *Commodities Market Speculation: The Risk to Food Security and Agriculture*. Minneapolis, MN: Institute for Agriculture and Trade Policy.

Jaffee D. 2007. *Brewing justice: Fair trade coffee, sustainability, and survival*. Berkeley, CA: University of California Press.

Jaffee D, and Howard PE. 2009. Corporate cooptation of organic and fair trade standards. *Agriculture and Human Values* **27**:387-99.

Jarvis D, Brown AHD, Cuong PH, *et al.* 2008. A global perspective of the richness and evenness of traditional crop-variety diversity maintained by farming communities. *Proceedings of the National Academy of Sciences* **105**: 5326-31.

Johns T and Eyzaguirre PB. 2006. Biofortification, biodiversity and diet: A search for complementary applications against poverty and malnutrition. *Food Policy* **32**: 1-24.

Jones, AD. 2015. The production diversity of subsistence farms in the Bolivian Andes is associated with the quality of child feeding practices as measured by a validated summary feeding index. *Public Health Nutrition*, **18**: 329-342.

Jones, AD, A Shrinivas, and R Bezner-Kerr. 2014. "Farm production diversity is associated with greater household dietary diversity in Malawi: Findings from nationally representative data. *Food Policy*, **46**: 1-12.

Kasem S and Thapa GB. 2011. Crop diversification in Thailand: Status, determinants, and effects on income and use of inputs. *Land Use Policy* **28**: 618–28.

Khan MA, Islam MR, Panaullah GM, *et al.* 2010. Accumulation of arsenic in soil and rice under wetland condition in Bangladesh. *Plant Soil* **333**: 263–74.

Klausmeyer KR, Shaw MR, MacKenzie JB, and Cameron DR. 2011. Landscape-scale indicators of biodiversity's vulnerability to climate change. *Ecosphere* **2**: art88.

Klooster D and Masera O. 2000. Community forest management in Mexico: carbon mitigation and biodiversity conservation through rural development. *Global Environmental Change* **10**: 259–72.

Lal R. 2009. Soil degradation as a reason for inadequate human nutrition. *Food Sec* **1**: 45–57.

Lappé FM, Clapp J, Anderson M, *et al.* 2013. How We Count Hunger Matters. *Ethics & International Affairs* **27**:251-59.

Leach M, Mearns R and Scoones I. 1999. Environmental entitlements: Dynamics and institutions in community-based natural resource management. *World Development* **27**: 225–47.

Lenzen M, Moran D, Kanemoto K, Foran B, Lobefaro L, and Geschke A. 2012. International trade drives biodiversity threats in developing nations. *Nature* **486**: 109-12.

Levidow L, Pimbert M and Vanloqueren G. 2014. Agroecological Research: Conforming—or Transforming the Dominant Agro-Food Regime? *Agroecology and Sustainable Food Systems* **38**: 1127–55.

Liebman M and Schulte LA. 2015. Enhancing agroecosystem performance and resilience through increased diversification of landscapes and cropping systems. *Elementa: Science of the Anthropocene* **3**: 000041.

Lira R, Casas A, Rosas-Lopez R, *et al.* 2009. Traditional Knowledge and Useful Plant Richness in the Tehuacan-Cuicatlan Valley, Mexico. *Econ Bot* **63**: 271–87.

Matson PA, Parton WJ, Power AG, and Swift MJ. 1997. Agricultural intensification and ecosystem properties. Science **277**:504-9.

McAfee K. 1999. Selling nature to save it? Biodiversity and green developmentalism. *Environment and Planning Development: Society and Space* **17**: 133–53.

McAfee K and Shapiro EN. 2010. Payments for Ecosystem Services in Mexico: Nature, neoliberalism, social movements and the state. *Ann Assoc Am Geogr* **100**: 579-599.

McDaniel MD, Tiemann LK, and Grandy AS. 2014. Does agricultural crop diversity enhance soil microbial biomass and organic matter dynamics? A meta-analysis. *Ecological Applications* **24**: 560–70.

McKean MA. 2000. Common Property: What is it, What is it good for, and What makes it work? In: Gibson CG, McKean M, Ostrom E (Eds). *People and Forests: Communities, Institutions, and Governance*. Cambridge: MIT Press.

McSweeney K. 2005. Indigenous Population Growth in the Lowland Neotropics: Social Science Insights for Biodiversity Conservation. *Conservation Biology* **19**: 1375–84.

Melo FPL, Arroyo-Rodríguez V, Fahrig L, *et al.* 2013. On the hope for biodiversity-friendly tropical landscapes. *Trends in Ecology & Evolution* **28**: 462–8.

Merenlender AM, Huntsinger L, Guthey G, and Fairfax SK. 2004. Land trusts and conservation easements: Who is conserving what for whom? *Conservation Biology* **18**: 65–76.

Mikkelson GM, Gonzalez A, and Peterson GD. 2007. Economic inequality predicts biodiversity loss. *PLoS ONE* **2**: e444.

Mozumder P and Berrens RP. 2007. Inorganic fertilizer use and biodiversity risk: An empirical investigation. *Ecological Economics*, **62**: 538-543.

Murphy S. 2009. *Strategic grain reserves in an era of volatility*. Minneapolis: Institute for Agriculture and Trade Policy.

Norton DA, Reid N and Young L. 2013. Ultimate drivers of native biodiversity change in agricultural systems. [v1; ref status: indexed, http://f1000r.es/21m] *F1000Research* **2**: 214

Nuñez-Espinoza JF and Tace-Nelson E. 2014. Nutrition and rural development policy in Latin America: The Human Nutrition Initiative. *Agricultura, Sociedad y Desarrollo* **11**: 125-51

Ó Gráda C. 2009. *Famine: A short history*. Princeton, NJ: Princeton University Press.

Ostrom EJ, Burger CB, Field RB, *et al* 1999. Revisiting the commons: Local lessons, global challenges. *Science* **284**: 278-82.

Otero G. 2011. Neoliberal Globalization, NAFTA and Migration: Mexico's Loss of Food and Labor Sovereignty. *Journal of Poverty* **15**: 384–402.

Otero G, Pechlaner G and Gürcan EC. 2013. The political economy of “food security” and trade: uneven and combined dependency. *Rural Sociology* **78**: 263–89.

Patel R, Bezner Kerr R, Shumba L and Dakishoni L. 2014. Cook, eat, man, woman: understanding the New Alliance for Food Security and Nutrition, nutritionism and its alternatives from Malawi. *Journal of Peasant Studies* **42**: 21-44.

Perfecto I and Vandermeer JH. 2008. Biodiversity conservation in tropical agroecosystems: a new conservation paradigm. *Annals of the New York Academy of Sciences* **1134**: 173–200.

Phelps J, Webb EL and Koh LP. 2011. Risky business: an uncertain future for biodiversity conservation finance through REDD+. *Conservation Letters* **4**:88-94.

Phelps J, Carrasco LR, Webb EL, *et al.* 2013. Agricultural intensification escalates future conservation costs. *Proceedings of the National Academy of Sciences* **110**: 7601–6.

Porter JR, Xie L, Challinor AJ, *et al*. 2014. Food security and food production systems. In: Field CB, Barros VR, Dokken DJ *et al.* (Eds). *Climate Change 2014: Impacts, Adaptation, and Vulnerability*. Cambridge, United Kingdom and New York, NY, USA: Cambridge University Press: 485-533.

Postma-Blaauw MB, de Goede RGM, Bloem J, *et al.* 2010. Soil biota community structure and abundance under agricultural intensification and extensification. *Ecology* **91**: 460–73.

Power AG. 2010. Ecosystem services and agriculture: tradeoffs and synergies. *Philosophical Transactions of the Royal Society of London B: Biological Sciences* **365:** 2959-71.

Raynolds LT. 2000. Re-embedding global agriculture: The international organic and fair trade movements. Agriculture and Human Values **17**: 297-309.

Regmi A and Meade B. 2013. Demand side drivers of global food security. *Global Food Security* **2**: 166–71.

Ringler C, Biswas AK, and Cline SA. 2010. *Global change: Impacts on water and food security*. Berlin; London: Springer.

Rocha C. 2009. Developments in national policies for food and nutrition security in Brazil. *Development Policy Review* **27**: 51–66.

Russell E. 2001. War and nature: fighting humans and insects with chemicals from World War I to Silent Spring. Studies in environment and history. Cambridge; New York: Cambridge University Press.

Savilaakso S, Laumonier, Y., Guariguata MR, and Nasi R. 2013. Does production of oil palm, soybean, or jatropha change biodiversity and ecosystem functions in tropical forests. *Environmental Evidence* **2**: 1–4.

Scherr, SJ. 1999. *Soil degradation: A threat to developing-country food security by 2020*?. Vol. 58. International Food Policy Research Institute.

Seed B, Lang T, Caraher M, and Ostry A. 2013. Integrating food security into public health and provincial government departments in British Columbia, Canada. *Agriculture and Human Values* **30**: 457–70.

Sen AK. 1981. *Poverty and famines: an essay on entitlement and deprivation*. Oxford, UK: Oxford University Press.

Seufert V, Ramankutty N, and Foley JA. 2012. Comparing the yields of organic and conventional agriculture. *Nature* **485**: 229–32.

Sievers-Glotzbach S. 2014. Reconciling intragenerational and intergenerational environmental justice in Philippine agriculture: The MASIPAG farmer network. *Ethic Pol Environ* **17**: 52–68.

Smith LC and Haddad L. 2000. *Explaining Child Malnutrition in Developing Countries; A cross-*

country analysis. Washington, DC: International Food Policy Research Institute.

Smith LC and Haddad L. 2015. Reducing Child Undernutrition: Past Drivers and Priorities for the Post-MDG Era. *World Development* **68**: 180–204.

Smith RJ, Muir RDJ, Walpole MJ, *et al*. 2003. Governance and the loss of biodiversity. *Nature* **426**: 67-70

Soares-Filho B, Rajao R, Macedo M, *et al.* 2014. Cracking Brazil's Forest Code. *Science* **344**: 363–4.

Staudt A, Leidner AK, Howard J, *et al.* 2013. The added complications of climate change: understanding and managing biodiversity and ecosystems. *Frontiers In Ecology And The Environment* **11**: 494–501.

Sumberg J, Keeney D, and Dempsey B. 2012a. Public Agronomy: Norman Borlaug as "Brand Hero" for the Green Revolution. *The Journal of Development Studies* **48**: 1587–600.

Sumberg J, Thompson J, and Woodhouse P. 2012b. Why agronomy in the developing world has become contentious. *Agriculture and Human Values* **30**: 71–83.

Tansey, G., and Rajotte T (Eds). 2008. *The Future Control of Food: A Guide to International Negotiations and Rules on Intellectual Property, Biodiversity and Food Security*. London: Earthscan.

Tilman D, Wedin J and Knops J. 1996. Productivity and sustainability influenced by biodiversity in grassland ecosystems. *Natur*e **379**: 718-720.

Turral H, Burke J and Faurès J. 2011. *Climate change, water and food security*. Rome: Food and Agriculture Organization of the United Nations.

Vandermeer JH, Lawrence D, Symstad A, and Hobbie SE. 2002. Effect of biodiversity on ecosystem functioning in managed ecosystems. In: Cotula L, Naeem S, Inchausti P (Eds). *Biodiversity and ecosystem functioning: Synthesis and perspectives*. Oxford: Oxford University Press.

Van Weelie D and Wals A. 2002. Making biodiversity meaningful through environmental education. *International Journal of Science Education* **24**: 1143–56.

Vanloqueren G and Baret PV. 2009. How agricultural research systems shape a technological regime that develops genetic engineering but locks out agroecological innovations. *Research Policy* **38**: 971–83.

Verburg PH, Mertz O, Erb K-H, *et al.* 2013. Land system change and food security: towards multi-scale land system solutions. *Current Opinion in Environmental Sustainability* **5**: 494–502.

Walker R. 2014. Sparing Land for Nature in the Brazilian Amazon: Implications from Location Rent Theory. *Geographical Analysis* **46**: 18–36.

Weinzettel J, Hertwich EG, Peters GP, *et al.* 2013. Affluence drives the global displacement of land use. *Global Environmental Change* **23**: 433–8.

Weis AJ. 2007. *The global food economy: The battle for the future of farming*. London and New York: Zed Books; Palgrave Macmillan.

White B, Borras SM Jr, Hall R, *et al.* 2012. The new enclosures: critical perspectives on corporate land deals. *Journal of Peasant Studies* **39**: 619–47.

Wise T. 2004. *The Paradox of Agricultural Subsidies: Measurement Issues, Agricultural Dumping, and Policy Reform*. Global Development and Environment Institute. Medford, MA: Tufts University.

Wise T. 2009. *Agricultural Dumping Under NAFTA: Estimating the Costs of U.S. Agricultural Policies to Mexican Producers*. Global Development and Environment Institute. Medford, MA: Tufts University.

Wittman H. 2009. Reframing agrarian citizenship: Land, life and power in Brazil. *Journal of Rural Studies* **25**: 120–30.

Wittman H. 2010. Agrarian Reform and the Environment: Fostering Ecological Citizenship in Mato Grosso, Brazil. *Canadian Journal of Development Studies* **29**: 281–98.

Wittman H. 2011. Food Sovereignty: A new rights framework for food and nature? *Environ Soc Adv Res* **2**: 87–105.

Wittman H and Blesh J. 2015. Food Sovereignty and Fome Zero: connecting public food procurement programs to sustainable rural development in Brazil. *Journal of Agrarian Change.* doi: 10.1111/joac.12131

Wittman H, Desmarais A, and Wiebe N (Eds). 2009. *Food Sovereignty in Canada: Creating Just and Sustainable Food Systems*. Halifax: Fernwood.

Wittman H, Desmarais A, and Wiebe N. 2010. *Food Sovereignty: Reconnecting Food, Nature and Community*. Halifax: Fernwood.

World Bank. 2007. *World Development Report 2008: Agriculture for Development*. Washington, DC: The World Bank.

Wright B. 2009. International Grain Reserves and Other Instruments To Address Volatility In Grain Markets. Washington, D.C.: World Bank.

Young A. 1999. Is There Really Spare Land? A Critique of Estimates of Available Cultivable Land in Developing Countries. *Environment, Development and Sustainability* **1**: 3–18.

Zedler JB. 2003. Wetlands at your service: reducing impacts of agriculture at the watershed scale. *Frontiers In Ecology And The Environment* **1**: 65–72.

Zimmerer KS. 1998. The ecogeography of Andean potatoes. *Bioscience* **48**: 445-454.

Zimmerer KS. Carney JA, and Vanek SJ. 2015. Sustainable smallholder intensification in global change? Pivotal spatial interactions, gendered livelihoods, and agrobiodiversity. *Current Opinion in Environmental Sustainability* **14**: 49–60.
